# The Prevalence and Predictors of Compassion Satisfaction, Burnout and Secondary Traumatic Stress in Registered Nurses in an Eastern Canadian Province: A Cross-Sectional Study

**DOI:** 10.1177/08445621221150297

**Published:** 2023-01-24

**Authors:** Joy Maddigan, Maureen Brennan, Kelly McNaughton, Gerry White, Nicole Snow

**Affiliations:** 1 Faculty of Nursing, Memorial University of Newfoundland and Labrador, St. John's, NL, Canada; 2 Organizational Development, Eastern Regional Health Authority, St. John's, NL, Canada; 3 Peer Support and Trauma Response Program, Toronto Hospital for Sick Kids, Toronto, ON, Canada; 4 Faculty of Medicine, Memorial University of Newfoundland and Labrador, St. John's, NL, Canada

**Keywords:** Nurses’ heath, worklife quality, work-related stress, job satisfaction

## Abstract

**Background:**

The quality of Registered Nurses’ worklife is impacting nurses’ mental health, and the standard of care received by clients. Contributing factors to nurses’ stress are the trauma of continuous caring for those in great suffering, and adverse working conditions.

**Objectives:**

i) to explore the prevalence of work-related stress in a provincial sample of Registered Nurses; ii) to compare the levels of compassion satisfaction, burnout and secondary traumatic stress reported by nurses in hospital, community, non-direct care settings, and, iii) to identify factors that predict levels of nursing work stress.

**Methods:**

A descriptive, predictive study with a self-report survey containing demographic questions and the Professional Quality of Life Scale was emailed to over 3,300 Registered Nurses. The scale measured the prevalence of three worklife indicators, compassion satisfaction, burnout and secondary traumatic stress. Multiple linear regression identified factors that predicted the levels of the three indicators. A subgroup analysis explored the quality of worklife based on three practice environments.

**Findings:**

Nurses (n  =  661) reported moderate compassion satisfaction, burnout, and secondary traumatic stress. The strongest predictor, satisfaction with one's current job, predicted high compassion satisfaction and lower burnout and secondary stress. The subgroup analysis identified hospital nurses as having the most work-related stress and the lowest level of compassion satisfaction.

**Conclusion:**

Innovative, collaborative action can transform nurses’ practice environments. Organizational support is essential to bring about needed improvements.

## Background and purpose

Many Registered Nurses (RNs) practice in environments that place them at risk for high levels of work-related stress and distress (Mooney et al., 2017; Wang et al., 2020). Of the health disciplines, nurses are identified as most affected by work stress (Favrod, et al., 2018; Stelnicki, et al., 2021; Woo, et al., 2020). This is due, in part, to the relational nature of nursing, which promotes nurses’ deep and meaningful connections with clients and coworkers (Hartrick Doane & Varcoe, 2015; Zhang et al., 2018). But relational practice can also deplete nurses’ resources as they continually intervene and bear witness to extreme suffering and sorrow (Djkowich et al., 2019; Hunsaker et al., 2015). Supporting nurses to manage the stress that is inherent in the practice of nursing is essential for the profession, and its members, to thrive.

Work-related stress has a profound effect on nurses’ well being, and the quality of their work life (Stelnicki & Carlton, 2021). The unrelenting and unpredictable nature of nurses’ work is a risk for unresolved work stress that can lead to clinical illnesses, such as, posttraumatic stress disorder, depression, and anxiety (Antony et al., 2020; O’Keefe et al., 2014). Workplace and organizational issues also contribute to stressful working conditions, particularly in acute care settings (Delgado et al., 2017; Hsiao et al., 2021, Kelly et al., 2021a). Factors related to high workloads, mandated overtime, bullying, physical violence, patient acuity and others, not only affect nurses’ health but also impact nurse retention and recruitment (Homburg et al., 2013; ten Hoeve et al., 2020), and contribute to the global nursing shortage (Dordunoo et al., 2021; Kristoffersen et al., 2016). Notably, outcomes of work-related stress go beyond the nursing workforce. The safety of clients and families is in jeopardy as nursing stress interferes with the delivery of quality nursing care, and constrains the effectiveness of the interdisciplinary team (Badu et al., 2020; Cavanagh et al., 2020; Cimiotti et al., 2012).

To support the wellbeing of nurses, the stress of nurses’ work must be monitored and successfully managed (Jakimowicz et al., 2018; Jappinen et al., 2021; Lopez-Lopez et al., 2019). Three quality of worklife indicators, compassion satisfaction (CS), burnout (BO), and secondary traumatic stress (STS), were developed to establish a greater understanding of the levels of work stress and work satisfaction of nurses (Figley, 1995; Stamm, 2010). Together, these indicators provide a interdependent, point-in-time picture of nurses’ quality of work life. CS, for example, reflects the joy and accomplishment that nurses have about the work they do, while burnout and STS represent negative impacts of nurses’ work-related stress (Stamm, 2010). Burnout is known by its gradual onset of hopelessness, ineffectiveness at work and disengagement. It is associated with a nonsupportive work environment (Stamm, 2010). STS, however, results from secondary exposure to individuals and families who have themselves experienced major traumatic events. It is a stress reaction characterized by fear, sleep disruptions, and avoidance behaviours (Stamm, 2010). The three indicators also represent more than a measure of the work stress experienced by nurses. They are indirect measures of organizational performance and the safety of the practice environment (Chu, 2021; Sarafis et al., 2016)

Since the early 1990’s, as working conditions in the health system deteriorated and nurses struggled to provide quality care, interest in nurses’ worklife and the indicators to measure it has steadily grown (Hemsworth et al., 2020; Schalk et al., 2010). New evidence is accumulating that points to the practice environment as having a greater impact on nurses’ stress levels than individual-level factors, and that organizational interventions produce longer lasting effects than individual level strategies (Bagnall et al., 2016). These findings highlight the importance of the organization and its responsibility for the active promotion of employee health, particularly when working conditions are a known contributor to ill health (Hollywood & Phillips, 2020). Undoubtably, work stress is a complex issue in health care and will require collaborative efforts and resources to ensure a healthy workplace for nurses.

A recent systematic review and meta-analysis (Xie et al., 2021) identifies current, evidence about the prevalence of work-related stress in Registered Nurses. It compares the stress levels of nurses in different regions of the world, providing valuable international context. In the review, 79 studies are analyzed to determine the levels of compassion satisfaction, burnout, and secondary traumatic stress in a combined sample of 28,509 nurses from 11 countries. The results reveal moderate levels of CS, BO and STS across the full study sample. When regional subgroups (Asia, Europe, Austraila and North America) are compared, differences in scores highlighted some notable trends (Xie et al., 2021). Nurses from Asia, for example, have both the highest work stress and lowest level of CS. Nurses from North Amercia have the highest level of CS but report the second highest level of work stress.

If progress is to be made on reducing the levels of work-related stress, Identification of key factors that influence prevalence is essential. Some factors, like job satisfaction (Homburg et al., 2013), resilience (Foster et al., 2020; Zhang et al., 2022), and supportive working relationships (Yu & Gui, 2021), are protective factors that have consistently demonstrated a positive influence on nurses’ work-related stress. Others, such as, being a novice nurse (Kawar et al., 2019), type of practice area (Kelly et al., 2015) and turnover intention (ten Hoeve et al., 2020) are risk factors that regularly predict lower levels of CS and higher BO. Both sets of factors play an important role in addressing the the problem, but much remains to be done. Evidence is lacking regarding the consistency and effect of other characteristics, such as age (Clari et al., 2021), gender (Mooney et al., 2017), and educational level (Kelly et al., 2021b). Additional research is needed to identify the factors that play a role in strengthening nurses’ wellbeing and quality of worklife (O’Callaghan et al., 2020).

This study aimed to establish the prevalence and severity of work-related stress in RNs in Newfoundland and Labrador by examining nurses’ professional quality of work life. No information was found that evaluated work life indicators for nurses in the study province. The identification and measurement of CS, BO, and STS are the first steps in bringing awareness to a potentially dangerous nursing workforce issue.

The three study objectives were: i) to explore the prevalence and severity of work-related stress in a diverse, provincial population of Registered Nurses in eastern Canada; ii) to compare the levels of compassion satisfaction, burnout and secondary traumatic stress reported by nurses from three practice environments: direct care hospital, direct care community, and non-direct care, and iii) to identify demographic and work factors that predict work-related stress levels for nurses.

## Methods

The study is the quantitative component of an explanatory sequential mixed methods investigation. It uses a cross-sectional design to examine work-related stress experienced by RNs and the factors that predict CS, BO, and STS.

### Participants and setting

Registered Nurses, including Nurse Practitioners, were recruited through the provincial regulatory body. Nurses who indicated a willingness to be contacted for research purposes on their 2019/20 nursing registration were the target sample. Just over 3,310 Registered Nurses, representing all regions of the province, received the electronic survey package by email in May 2019. Study recruitment was open for 16 weeks.

### Measures

Work-related stress is measured using the well validated Professional Quality of Life Scale (ProQOL5; Stamm, 2010; O’Callaghan et al., 2020). ProQOL is a 30-item questionnaire made up of three subscales, CS, BO and STS. The subscales, each with 10-items, provide information about the level of work stress experienced by nurses and its impact on nurses’ psychological well-being. Each item is scored on a Likert Scale ranging from 1 (never) to 5 (very often). Total scores for each subscale range between 10 and 50. Based on scoring recommendations (Stamm, 2010), scores on the three subscales are categorized as low [scores of 22 or less], moderate [scores between 23–41] and high [scores of 42 and over]. High scores for CS represent a positive health state while high scores for BO and STS indicate a negative health state. Stamm (2010) reports that the reliability of the three subscales ranged from 0.84 to 0.90. ProQOL demonstrated good reliability in the current study with Cronbach's alpha at 0.91 for CS, 0.84 for BO, and 0.87 for STS.

Eleven demographic and occupational variables provide information about the sample. Age is collected in 10-year age categories starting at 20 to 29 years of age. Gender is collected as male, female, other, or prefers not to answer. Information on current employer includes the four regional health authorities that provide the publicly funded health services in the province, and other. One question measures the level of job satisfaction as ‘not satisfied’, ‘satisfied’ and ‘very satisfied’, and one question measures the participants intention to leave their job within the next 12 months as ‘yes’, ‘no’ or ‘undecided. The six remaining variables include years as an RN, nurse practitioner designation, level of education, current practice environment, worked hours per week and number of patients assigned last shift.

### Data collection

The electronic survey was emailed to Registered Nurses by the regulatory body. The ProQOL survey and demographic questionnaire were included in the survey package. The returned survey was anonymous and could not be tracked back to the sender. A survey reminder was sent during the third week of active recruitment and again during week ten of the recruitment period.

### Data analysis

Data were analysed using the Statistical Package for the Social Sciences (IBM SPSS Statistics for Windows, version 27). Demographic and occupational variables were measured using descriptive statistics. Frequencies, means, standard deviations, medians, and ranges were calculated as appropriate. Bivariate analyses were carried out to examine relationships between the independent and dependent variables. One-way analysis of variance (ANOVA) examined associations among the demographic and work variables and CS, BO and STS (supplemental data available on request). The α was .05. Multiple linear regression (MLR) was used to identify the independent variables that predicted the levels of CS, BO and STS. With nine independent variables for the MLR, the study's minimum sample size was 180 participants (20 per variable) to achieve 95% power and a medium effect size (.15) at α  =  .05 (Green, 1991; Hunsaker et al., 2015). The nine variables were entered into the regression model simultaneously.

### Ethics

Participants who opened the electronic survey were provided an information letter explaining the study and a contact number for the principal investigator if they had questions. Included with the survey was a list of counselling resources, in case participants experienced any distress resulting from the study questions. The study received ethics approval (HREB #20192165).

## Results

Questionnaires were returned by 810 nurses for a total response rate of 24.5%. Surveys with more than 10% of items omitted were removed from the analysis, resulting in a study sample of 672 participants and a response rate of 20.4%.

### Characteristics of registered nurses

Six hundred and seventy-two Registered Nurses provided demographic and occupational information (see Table 1 below). The majority (94.2%, n  =  633) were female and 36.6% (n  =  246) were under 40 years of age. Nurses between 40 and 49 years made up 30.7% (n  =  206) of participants while 31.9% (n  =  220) were 50 years or older. Nurses with fewer than six years of nursing experience comprised 15.2% (n  =  102) of the sample. Most nurses (72.5%, n  =  487) had worked as an RN for more than 10 years. Over three-quarters of the respondents (76.5%, n  =  514) worked full time and the majority (89.7%, n  =  603) were employed in the provincial health system, that is, with one of the four regional health authorities. Based on their current nursing position, nurses were categorized into one of three practice environments: i) ‘direct care hospital’ nurses comprised 53.1% (n  =  351) of study participants; ii) ‘*direct care community’* nurses made up 31.8%,(n  =  210) and iii) nurses working in ‘*non-direct care’*, such as education or administration, comprised 15.1% (n  =  100) of all participants. Most nurses were satisfied (58.5%,n  =  398) or very satisfied (18.2%, n  =  122)) with the work they were doing. Nearly one-quarter (23.4%, n  =  157), however, indicated dissatisfaction with their present job situation. While the majority of nurses (57.3%, n  =  384) had no intention of leaving their position in the next year, 90 nurses (13.4%) reported that they had made the decision to leave their present job. In addition, 197 nurses (29.4%,) reported they were uncertain as to whether they would leave their current job.

**Table 1.** Demographic and occupational characteristics of registered nurses.

Characteristic	Criteria	Number	Percent
Age Group (n = 672)	20–29 years	102	15.2%
	30–39	144	21.4
	40–49	206	30.7
	50–59	174	25.9
	60 and Older	46	6.8
Gender (n =** ** 672)	Female	633	94.2%
	Male	33	4.9
	Prefer not to answer	6	0.9
Education (n =** ** 662)	RN diploma	200	29.8%
	Bachelor degree	373	55.5
	Graduate degree	89	14.2
Years as a Nurse (n =** ** 672)	5 & under	102	15.2%
	6–10 y	83	12.4
	11–20 y	167	24.9
	21–30 y	180	26.8
	30 & over	140	20.8
NP Designation (n =** ** 36)	Working as an NP	28	77.8%
Employer (n =** ** 672)	Eastern Health	375	55.8%
	Central Health	70	10.4
	Western Health	109	16.2
	Labrador-Grenfell	49	7.3
	Other	69	10.2
Practice Environment (n =** ** 661)	Direct care hospital	351	53.1%
	Direct care community	210	31.8
	Non-direct care	100	15.1
Wkly.Work Hours (n =** ** 670)	24 h or less	45	6.7%
	25–36 h	111	16.5
	37–50 h	488	72.6
	Over 50 h	26	3.9
Pt Assigned Last Shift (n =** ** 348)	3 or less pts	81	23.3%
	4–6 pts	167	48.0
	7–15 pts	42	12.1
	Over 15 pts	58	16.7
Job Satisfaction (n =** ** 672)	Not satisfied	157	23.4%
	Satisfied	393	58.5
	Very satisfied	122	18.2
Turnover Intention (n =** ** 671)	Yes	90	13.4%
	No	384	57.3
	Undecided	197	29.3

### Prevalence and severity of work-related stress

#### Results for study objective 1

##### Total provincial sample

Mean scores on the ProQOL subscales indicated that nurses in this study experienced moderate levels of CS, BO and STS. The mean score for CS was 36.97 (SD  =  6.45); the range was 36 (14–50). For BO, the mean score was 26.29 (SD  =  6.08) and the range was 31 (11–42). For STS, the mean score was 23.81 (SD  =  6.52), and the range was 37 (10–47). Median scores also indicated moderate levels of quality of life, with CS at 38, BO at 26 and STS at 23.

**Figure 1.**
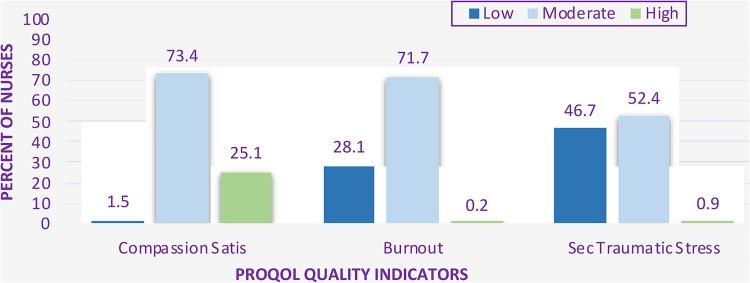
Distribution of ProQOL subscale scores.

Over 98% of participants demonstrated moderate to high levels of positive compassion satisfaction with just ten nurses reporting low levels (see Figure 1). The negative effects of burnout were felt by many nurses, as over two-thirds (71.1%) reported a moderate level of BO. Secondary traumatic stress had a lesser impact with just over half the nurses reporting a moderate level of STS; only six nurses (less than 1%) reported high secondary traumatic stress levels.

#### Results for study objective 2

##### Three practice environments

**Figure 2.**
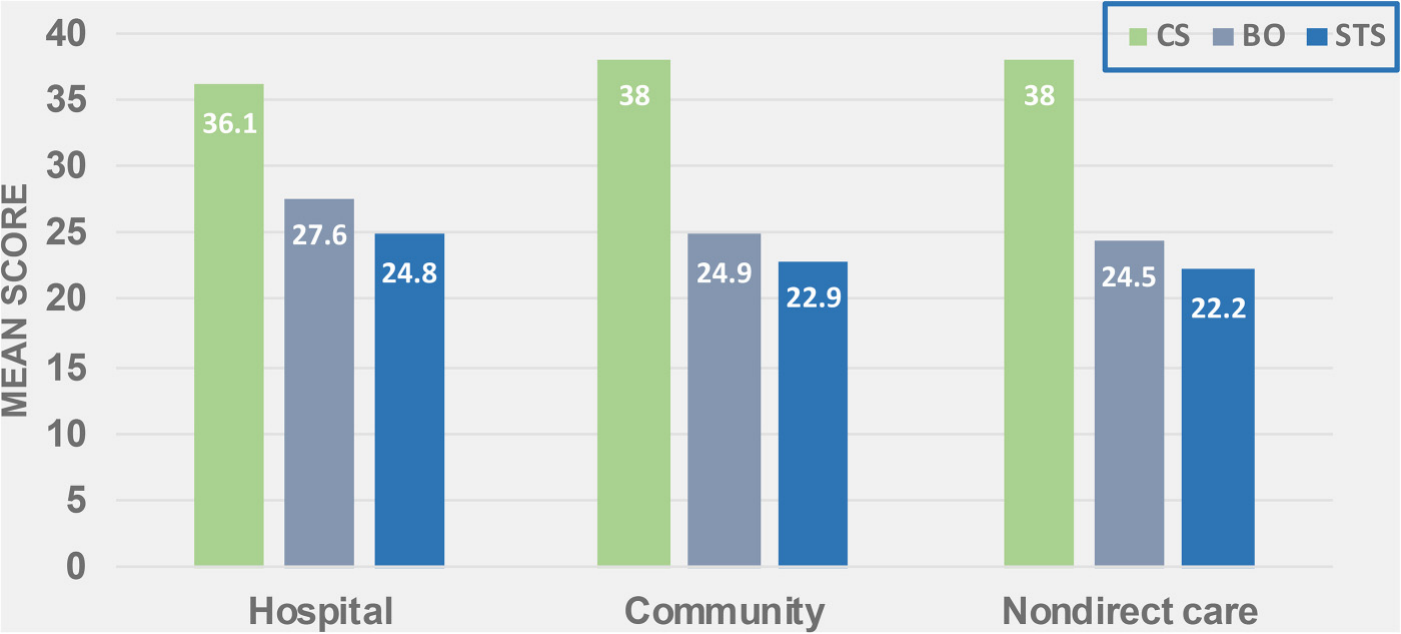
Distribution of ProQOL mean scores by practice environment.

When examined as separate groups, direct care hospital nurses (n  =  351), direct care community nurses (n  =  210) and non-direct care nurses (n  =  100) each reported moderate levels of CS, BO and STS. The mean scores and ranges for the three indicators reported by hospital nurses are: i) CS: mean  =  36.12 (SD  =  6.34) with a range of 35 (15–50); ii) BO: mean  =  27.58 (SD  =  5.94); range  =  30 (12–42), and iii) STS: mean  =  24.77 (SD  =  6.41); range  =  36 (11–47). For community Registered Nurses who work in areas such as mental health and addictions, community health, and long term care, the mean scores and ranges are: i) CS: mean  =  38.01 (SD  =  6.32), range  =  36 (14–50): ii) BO: mean  =  24.91 (SD  =  5.86), range  =  27 (11–38); iii) STS: mean  =  22.90 (SD  =  6.18), range  =  30 (10–40). For the third and smallest practice area representing nurses providing other than direct care, scores and ranges reported for the three indicators are: i) CS: mean  =  38.03 (SD  =  6.32); range  =  32 (18–50); ii) BO: mean  =  24.53 (SD  =  5.81); range  =  28 (11–39): iii) STS: mean  =  22.18 (SD  =  6.74); range  =  36 (10–46) (Figure 2).

### Factors that predict work-related stress in registered nurses

#### Results for study objective 3: provincial sample

##### Total sample multiple linear regressio*n*

MLR findings revealed that both similar and unique variables predicted the scores on the three ProQOL subscales. Two independent variables, i) being satisfied with one's current job, and ii) being undecided about whether or not to stay in one's current position were predictors across the three subscales. The level of job satisfaction had the highest impact of the nine independent variables on each subscale (β  =  .432, p < .000 for CS; β  =  -.462, p < .000 for BO; and β  =  -.286, p < .000 for STS). It is the strongest predictor of CS, BO and STS for the study population. 

##### Compassion satisfaction

Four variables were significant predictors of compassion satisfaction. Job satisfaction (β  =  .432, p < .000) and being 50 years or more (β =  .160, p < .000) are predictive of higher levels of CS while nurses under 30 years (β  =  -.102 p <.006) and those who were uncertain about staying in their current position (β  =  -.147, p < .000) had lower CS scores. In combination, these four variables explained 30% of the variance in levels of CS.

##### Burnout

For the six significant predictors of burnout, four were similar to those that predicted CS levels and two, i) hours worked per week and ii) a confirmed intention to leave one's job within 12 months, were different. Older age (β  =  -.171, p < .000), and job satisfaction (β  =  -.462, p < .000) predicted lower burnout scores while the remaining four variables predicted higher burnout. Uncertainty about staying in one's current job was the strongest predictor of higher burnout (β  =  .138, p < .000), followed by working fulltime hours (β  =  .117, p < .000). Younger nurses (β = 112, p < .002), and nurses who had decided to leave their current job (β  =  .073, p <.033) also had higher burnout. The six variables accounted for 35% of the variance in burnout scores.

##### Secondary traumatic stress

Five significant predictors influenced STS. Being male (β  =  -.091, p <.015) and being satisfied with one's job (β  =  -.286, p <.000) predicted lower STS scores. Being employed full time (β  =  .098, p <.009), having made the decision to leave one's job (β  =  .078, p < .049), and being uncertain about leaving one's current position (β  =  .110. p < .005) made a weak contribution to higher STS scores. The five variables predicted 14% of the variance found in the STS scores (Table 2).

**Table 2.** Factors that predict stress levels in registered nurses (n  =  661).

Variables	Compassion Satisfaction: Subscale 1			Burnout: Subscale 2			Secondary Traumatic Stress: Subscale 3		
	SE B	Std. *B*	t	SE B	Std. *B*	t	SE B	Std. *B*	t
Constant	4.199		7.537	3.788		7.860	4.683		7.620
Gender	1.003	.011	.331	.905	−.017	−.538	1.120	**−**.091*	−2.430
Age−young	.553	−.102*	−2.766	.498	.112*	3.175	.620	.045	1.090
Age−old	.538	.160*	4.135	.485	**−**.171*	−4.618	.603	−.024	−.566
RN−BN	.679	−.025	−.482	.606	.020	.396	.754	−.063	−1.014
RN−diploma	.727	−.047	−.909	.647	.027	.561	.808	−.080	−1.399
Worked hrs/ wk	.519	−.011	−.317	.464	.117*	3.619	.575	.098*	2.611
Job satisfaction−yes	.533	.432*	12.306	.483	**−**.462*	−3.710	.597	**−**.286*	−7.359
Intention to leave−yes	.674	−.050	−1.412	.612	.073*	2.131	.769	.078*	1.947
Int to leave−uncertain	.508	** −**.147*	−4.115	.456	.138*	4.034	.564	.110*	2.790
*p<0.05	Note: r = .544, r^2^ = .296 **(30%)** r^2adjusted^ = .286			Note: r = .590, r^2^ = .346, **(35%)** r^2adjusted^ = .339			Note: r = .371 r^2^ = .137, **(14%)** r^2adjusted^ = .125		

#### Results for study objective 3: subgroups

##### Subgroup multiple linear regressions

Multiple linear regression (MLR) was also conducted to identify the occupational and demographic predictors for direct care nurses in hospital and community environments (Table 3). Due to an insufficient number of participants, MLR was not conducted on the non-direct care group.

**Table 3.** Factors that predict stress in hospital and community direct care nurses

Variables	Direct Care Hospital Settings						Direct Care Community Settings					
	Compassion Satisfaction		Burnout		Secondary Traumatic Stress		Compassion Satisfaction		Burnout		Secondary Traumatic Stress	
	**β** [SE B]	*t*	**β** [SE B]	*t*	**β** [SE B]	*t*	**β** [SE B]	*t*	**β** [SE B]	*t*	**β** [SE B]	*t*
Constant	− [5.286]	5.966	− [4.768]	5.921	− [6.129]	5.832	− [11.001]	1.727	− [9.198]	3.767	− [11.127]	2.754
Gender	.032 [1.232]	.713	−.038 [1.111]	−.865	−.115* [1.426]	−2.191	.081 [2.686]	1.234	−.031 [2.246]	−.500	−.027 [2.723]	−.391
Age Under 30y	−.177* [.679]	−3.419	.204* [.611]	4.097	.089 [.790]	1.490	−.006 [1.352]	−.093	−.119 [1.130]	−1.856	−.167* [1.372]	−2.283
Age 50y +	.143* .814	2.609	−.117* [.743]	−2.209	.032 [.954]	.509	.183* [.993]	2.476	−.311* [.824]	−4.491	−.086 [1.006]	−1.076
RN−BN	−.132 [1.221]	−1.398	.246* [1.101]	2.724	−.005 [1.440]	−.044	−.039 [1.383]	−.371	−.121 [1.128]	−1.244	−.088 [1.368]	−.797
RN−diploma	−.197* [1.304]	−2.034	.253* [1.178]	2.721	−.050 [1.550]	−.439	−.052 [1.482]	−.497	−.068 [1.195]	−.716	−.059 [1.449]	−.542
Worked hrs. per week	−.012 [.654]	−.277	.141* [.591]	3.251	.127* .763	2.436	.000 [1.084]	−.005	.111 [.884]	1.738	.048 [1.065]	.656
Satisfaction with job − yes	.464* [.648]	9.958	−.481* [.587]	−10.749	−.264* [.756]	−4.927	.386* [1.194]	5.450	−.418* [.992]	−6.359	−.231* [1.190]	−3.079
Intention to leave − yes	−.099* [.861]	−2.074	.115* [.778]	2.495	.135* [1.008]	2.450	.031 1.313	.448	−.046 [1.114]	−.698	−.082 [1.373]	−1.103
Intention to leave − undecided	−.164* [.652]	−3.450	.160* [.588]	3.502	.093 [.757]	1.695	−.072 [1.051]	−.990	.040 [.864]	.588	.069 [1.041]	.903
*p<0.05	Note: r = .578, r^2^ = .334 **(33%),** r^2adjusted^ = .316		Note: r = .622, r^2^ = .387 **(39%),** r^2adjusted^ = .371		Note: r = .363, r^2^ = .132, **(13%)** r^2adjusted^ = .108		Note: r = .477, r^2^ = .228**, (23%)** r^2adjusted^ = .190		Note: r = .559, r^2^ = .312 **(31%)** r^2adjusted^ = .279		Note: r = .329, r^2^ = .108 **(11%),** r^2adjusted^ = .065	

##### Direct care hospital nurses (n  =  345)

Two factors predicted higher levels of CS. Nurses who were 50 years and older (β  =  .143, p <.05) and those who reported job satisfaction (β  =  .464, p < .05 had higher CS levels. Four predictors had a negative impact on CS levels: i) age under 30 years (β =  -.177, p < .05), ii) having an RN diploma (β  =  -.197, p < .05), iii) having made the decision to leave one's current position (β  =  -.099, p <.05), and iv) being undecided about whether to leave one's position (β  =  -.164, p < .05). In total, 33% of the variance in CS scores is attributed to the six significant independent variables.

Burnout was affected by eight of the nine independent variables (Table 3). Gender was not a significant predictor. The two variables that enhanced CS, older age (β  =  -.117, p <.05) and positive job satisfaction (β  =  -.481. p < .05), were also protective against BO. Six variables were found to increase levels of BO. Nurses who intended to leave their job (β  =  .115, p < .05), or were uncertain about staying in their job (β  =  .160, p < .05) had higher levels of BO. Nurses of younger age (β  =  .204, p < .05); those working full time hours (β  =  .141, p < .05), and those having either an RN diploma (β  =  .253, p < .05) or a bacaulaurate degree (β  =  .246, p <.05) also had heightened BO scores. Thirty-nine percent of the variance in burnout levels resulted from the eight independent variables.

Four variables were weak predictors of STS. Being male (β  =  -.115, p < .05) and having positive job satisfaction (β =  -.264, p <.05) predicted lower STS scores. Two variables, full time work (β  =  .127, p <.05) and a decision to leave one's job (β  =  .135, p < .05) predicted higher scores. In total, 13% of the variance in STS scores can be attributed to these variables.

##### Direct care community nurses

For nurses with a community practice, demographic and occupational characteristics were significant, but not strong predictors of CS, BO, or STS levels (Table 3). Two exceptions were the variables, job satisfaction and age. Satisfaction with one's job predicted higher CS (β  =  .386, p < .05), lower BO (β  =  -.418, p < .05) and lower STS (β  =  -.231, p < .05) for community nurses. Older age predicted higher CS scores (β  =  .183, p < .05) and lower burnout (β  =  -.311, p < .05) while younger age was predictive of higher STS (β  =  -.167, p <.05). For CS, 23% of the variance in scores was due to the two predictors. Age and job satisfaction also predicted 31% of the variance in BO scores and 11% of the variance in STS.

## Discussion

### Prevalence of CS, B0, STS

This study is the first known exploration of the prevalence and predictors of work-related stress in Registered Nurses in Newfoundland and Labrador. One of the main study findings, mean scores for compassion satisfaction, burnout and secondary traumatic stress as measures of nurses’ day-to-day work stress, identified moderate levels of CS, BO and STS in the diverse, provincial sample (Xie et al., 2021). The moderate mean scores for the three measures suggest that, for the majority of nurses, work-related stress, while clearly felt, was not at a high level at the time the study was conducted. Many nurses were successful in addressing the work stress they experience. However, when the scoring range for each measure is examined for variability, CS and STS had wider ranges indicating more extreme scores (low and / or high) than the scores found for burnout. This may point to a number of nurses in the study who are affected by high levels of work-related stress and low levels of CS but remain somewhat invisible within the larger group.

The prevalence of work stress in the health system is pervasive. Over 70% of study participants reported moderate levels of burnout, while 29% reported low levels. The large number of nurses experiencing symptoms of burnout is concerning as burnout poses a risk to both nurses’ health and professional abilities (Woo, et al., 2020). Moderate burnout is identified when nurses experience some, but not all, the symptoms of BO and the symptoms are not at a level where clinical intervention is required (Stelnicki et al., 2021). Feelings of emotional exhaustion, cynicism, negativity and a decreased sense of personal accomplishment are common manifastations of burnout (WHO, 2019). The severity of these symptoms determimes the severity of burnout (Stelnicki, et al., 2021). STS had a lower prevalence than BO. Fifty-two percent of nurses reported moderate levels of STS and 48% reported low levels of STS. Fewer nurses were affected by the stress of secondary trauma and, if they were affected, the level of stress was not as severe as was seen with BO. This finding is consistent with Bock et al. (2020), as they found that 91% of the 320 nurses in their sample experienced secondary trauma events with 25.3% (n  =  74) having secondary traumatic symptoms.

The prevalence of compassion satisfaction in this study is higher than the prevalence of BO or STS. Nurses reported moderate (73%) and high (25%) levels of CS, which suggests the nursing profession remains a source of joy and fulfillment for most nurses. Compassion satisfaction is known to have a positive influence on nurses’ quality of worklife (Chen et al., 2021; Figely, 1995) and may be a protective factor in the management of work-related stress (Lee et al., 2021). These findings reflect the underpinnings of Stamm's conceptual model regarding the interconnectness of the three worklife measures (Stamm, 2010). While each measure is separate, they have to be examined together to understand the full impact of work-related stress.

### CS, BO and STS by three practice environments

For nurses in the three practice environments, closer examination of work-related stress identified a consistent trend. Although the three groups report moderate levels of CS, BO and STS, direct care hospital nurses experience higher levels of burnout and secondary traumatic stress than either direct care community nurses or nurses working in non-direct care. They also report lower levels of compassion satisfaction. This pattern of scores suggests that, for nurses, the highest level of work stress is found in the secondary and tertiary components of the health system. Evidence suggests that patient acuity, including regular admissions resulting from a range of person-related traumas, is a unique contributor to the stress felt by hospital-based nurses (Lowe et al., 2021).

Nurses working directly with clients in the community present a different profile of quality of worklife measures compared with hospital nurses but similar to nurses working in nondirect care. Community and nondirect care nurses demonstrated higher CS and lower BO and STS than nurses working in a hospital environment. This pattern of scores is consistent with findings described by Durkin et al. (2016). They report that when community nurses’ compassion satisfaction and self-compassion are high, the nurses experience a better quality of worklife and are more resilient in dealing with work-related stress. Dor et al. (2019) reported the same trend for burnout in community nurses.

### Predictors of work-related stress in nurses

A range of predictors in this study influenced nurses’ quality of worklife. Knowledge of predictors provides key directions for reducing the prevalence of BO and STS and increasing the prevalence of CS. They are important for effective workplace improvements. The most compelling predictor identified in this study was the effect of job satisfaction on the three workplace measures. Job satisfaction is consistently recognized as a critical factor for a strong, healthy nursing workforce (Lee et al., 2021). In our study being satisified with one's job strengthened CS and lowered levels of BO and STS. A second strong predictor of severity of work related stress was the intention to leave one's current position within the next 12 months. Evidence is accumulating that work stress is an important factor in nurse's Intention to leave a job (Kelly et al., 2021; Kim & Kim, 2021). In our study, lower CS and higher BO and STS were predicted when nurses reported indecision about whether or not to leave their current position. The age of the nurse was also a frequent predictor of CS and BO, but not STS, in our study. Older nurses had higher CS and lower BO than other nurses while younger nurses experienced less CS and higher burnout. Kawar et al. (2019) found similar results in relation to age. The number of hours worked was a significant predictor for BO and STS; nurses who worked at least full time hours had higher levels of both BO and STS than nurses working part time. This study finding is supported in the literature (Wang et al., 2020).

#### Predictors and practice environments

Predictors for direct care hospital nurses were similar to the predictors identified for the total sample, but also included additional significant predictors. For example, low compassion satisfaction was impacted by four variables; two more than the total sample. Being a diploma-prepared nurse, and having made the decision to leave their job, were also predictive of lower CS for hospital nurses. Levels of burnout were predicted by eight of the nine variables, with gender as the only exception. These variables explained 39% of the variance in burnout scores. According to Zaghini et al. (2020) high levels of emotional labor and work stress increased BO levels in nurses with work stress mediating the relationship between emotional labor and BO. Although four significant variables were are predictors for STS, they accounted for only 13% of the variance in STS levels. Other, more targeted predictors are needed for STS.

The same may be true for nurses who work in the community. Current predictors have focused on the acute care environment and may not reflect the nature of their work (Hu et al., 2015) Two exceptions in our study are job satisfaction and age. Similar to hospital nurses, satisfaction with one's job predicted higher CS and lower work-related stress (BO and STS). Older age predicted higher CS and lower BO while nurses of a younger age tended to have higher levels of STS. Twenty -three percent of the variance in CS levels, 31% of the variance in BO and 11% of the variance in STS are attributed to job satisfaction and age.

### Study implications

Study findings can be used as a catalyst for needed change in the provincial health system. In the immediate, the information can help increase nurses’ awareness of the work-related risks present in the workplace. It can can also alert health administrators to critical workplace risk factors that can be successfully measured and addressed (Gibb et al., 2010; Ren et al., 2020). The adverse issues that stem from an unhealthy workplace are many and and can escalate into serious clinical disorders for some nurses (Lauvrud, et al., 2009). Without a good understanding of the problem and consistent efforts to monitor and respond to workplace risks, nursing work stress will continue to develop and negatively impact nurses, clients and the health organization (Jun et al., 2021). One needed response is to increase intervention research to test the effect of specific strategies and policies within the practice environment (Kelly et al., 2021). Individual level programs such as resilience training (Zhang, et al., 2022) and cognitive emotional regulation (Chang & Shin, 2021) are known to reduce work stress. Organizational strategies are often more powerful (Jun, et al., 2021) and new models of nursing are rrecommended to address workforce issues and prepare nurses to meet current day challenges (Jackson et al., 2022).

From a broader perspective, deeper, more sustainable change is needed if nurses are to optimize their influence in the practice area. The human experiences that nurses encounter on a daily basis will continue to bring both joys and sorrows, moral and ethical dilemmas and everyday problems to be solved. Experiencing stress and traumatic events are the norm for most nurses and they have to be accepted as one of the challenges of the profession (Clari et al., 2021). This reality does necessitate, however, that nurses and nursing students be well equipped and well supported to successfully cope and grow from the challenges that are inherent in the work of nurses.

A number of large scale initiatives have been developed to respond to nurses and other health employees to increase their engagement with work. Quadruple Aim is a framework used by health organizations to guide health system performance (Bodenheimer & Sinsky, 2015). Developed by the Institute for Health Improvement in the United States, it identifies provider wellness, including finding meaning and joy in work, as a critical component of health system performance (Sikka et al., 2015). Magnet hospitals are another example of a major hospital redesign that focuses on a healthy work environment for nurses (Petit Dit Dariel & Regnaux, 2015). Accreditated by the American Nurses Credentialing Centre, hospitals world wide are creating significant changes in their operations to achieve the standards that have been established for optimal nursing performance. Involving direct care nurses in all levels of organizational decision making is one example of a Magnet requirement.

Nursing education's role in the selection and preparation of students to meet the challenges of practice is key to a healthy nursing workforce. Accurate information for potential applicants and the use of best practices in admission assessments are essential aspects of a successful intake of students (Zamanzadeh et al., 2020). Undergraduate nursing programs need to be more active in supporting the mental health of students and initiate strategies to reduce stigma and discrimination toward students living with mental illness. Adoption of the ‘National Standard for Mental Health and Well-Being in Post-secondary Students’ (Mental Health Commission of Canada, 2020) in nursing faculties would provide direction for best practices in supporting and strengthening student mental health.

Nurses who are new to practice also face obstacles in the first years of employment (Hallaran et al., 2021; Duchscher, 2009). Successful mentorship relationships are proven to facilitate a smooth transition of new nurses into the workplace (Weng et al., 2010). Other strategies, such as ensuring supervisors connect with and support new nurses (Yu & Gui, 2021), and implementation of program policies that prioritize the mental health and psychological safety of nurses can help establish a healthy workplace climate.

### Study limitations and strengths

Cross-sectional studies are weak, descriptive designs that do not address causal relationships. A convenience sample was recruited and only self-report data were collected. Both factors introduce bias in the study results. The low survey response rate of 20.4% was also a study limitation. Although the sample size had adequate power to detect significant differences among the predictors, its generalizability is limited. Use of one instrument to collect data on three complex indicators also limited the findings. Study strengths include the collection and reporting of novel, quality worklife indicators that have not been previously examined. Other strengths are the provincial sample, the broad range of the nursing work that is represented, and the variety of locations and settings from which the sample was drawn.

## Conclusion

This study provided insight into the breadth and depth of the work-related stress experienced by Registered Nurses in Newfoundland and Labrador just prior to the COVID-19 pandemic. Measures of burnout and secondary traumatic stress identified moderate levels of work stress among participants which were juxtaposed with moderate levels of compassion satidsfaction This suggests that, despite negative working conditions, nurses continued to experience meaning and satisfaction in their work. The high number of nurses who experience symptoms of burnout and those who are also affected by stress related to traumatic events are evidence of the price that nurses pay to nurse. It cannot be overlooked. Direct care nurses who worked in a hospital setting experienced a lower quality of worklife than either community nurses or nurses who worked in non-direct care. Workplace improvements, led by nurses, are needed for nurses to excel in their roles. A comprehensive, collaborative approach is required to positively affect the quality of nurses’ worklife and protect their mental health.
